# The global aHUS registry: methodology and initial patient characteristics

**DOI:** 10.1186/s12882-015-0195-1

**Published:** 2015-12-10

**Authors:** Christoph Licht, Gianluigi Ardissino, Gema Ariceta, David Cohen, J. Alexander Cole, Christoph Gasteyger, Larry A. Greenbaum, Sally Johnson, Masayo Ogawa, Franz Schaefer, Johan Vande Walle, Véronique Frémeaux-Bacchi

**Affiliations:** Division of Nephrology and Program in Cell Biology, The Hospital for Sick Children, 555 University Avenue, Toronto, ON M5G 1X8 Canada; Fondazione IRCCS Ca’ Granda Ospedale Maggiore Policlinico, Via della Comenda 9, Milan, 20122 Italy; Pediatric Nephrology, University Hospital Vall d’Hebron, Pg Vall d’ Hebron, 119-129 Barcelona, Spain; Columbia University Medical Center, 622 West 168 Street, Room PH4-124, New York, NY USA; Alexion Pharmaceuticals, Inc., 352 Knotter Drive, Cheshire, CT USA; Alexion Pharma International, Avenue du Tribunal Fédéral 34, Lausanne, Switzerland; Emory University and Children’s Healthcare of Atlanta, 2015 Uppergate Drive, Atlanta, GA USA; Great North Children’s Hospital, Sir James Spence Institute, 4th floor, Royal Victoria Infirmary, Newcastle, United Kingdom; Heidelberg University Medical Center, Im Neuenheimer Feld 672, 69120 Heidelberg, Germany; Ghent University Hospital, De Pintelaan 185, Ghent, Belgium; Assistance Publique-Hôpitaux de Paris, Hôpital Européen Georges Pompidou, 20 rue Leblanc, Paris, France

**Keywords:** Complement, Hemolytic uremic syndrome, Renal insufficiency

## Abstract

**Background:**

Atypical hemolytic uremic syndrome (aHUS) is a rare, genetically-mediated systemic disease most often caused by chronic, uncontrolled complement activation that leads to systemic thrombotic microangiopathy (TMA) and renal and other end-organ damage.

**Methods:**

The global aHUS Registry, initiated in April 2012, is an observational, noninterventional, multicenter registry designed to collect demographic characteristics, medical and disease history, treatment effectiveness and safety outcomes data for aHUS patients. The global aHUS Registry will operate for a minimum of 5 years of follow-up. Enrollment is open to all patients with a clinical diagnosis of aHUS, with no requirement for identified complement gene mutations, polymorphisms or autoantibodies or particular type of therapy/management.

**Results:**

As of September 30, 2014, 516 patients from 16 countries were enrolled. At enrollment, 315 (61.0 %) were adults (≥18 years) and 201 (39.0 %) were <18 years of age. Mean (standard deviation [SD]) age at diagnosis was 22.7 (20.5) years. Nineteen percent of patients had a family history of aHUS, 60.3 % had received plasma exchange/plasma infusion, 59.5 % had a history of dialysis, and 19.6 % had received ≥1 kidney transplant. Overall, 305 patients (59.1 %) have received eculizumab.

**Conclusions:**

As enrollment and follow-up proceed, the global aHUS Registry is expected to yield valuable baseline, natural history, medical outcomes, treatment effectiveness and safety data from a diverse population of patients with aHUS.

**Trial registration:**

US National Institutes of Health www.ClinicalTrials.gov Identifier NCT01522183. Registered January 18, 2012.

## Background

Atypical hemolytic uremic syndrome (aHUS) is a rare, genetic, life-threatening systemic disease that has an incidence of one to two cases per million [1, 2]. It is most often caused by chronic, uncontrolled activation of the complement system, which leads to activation of endothelial cells, recruitment of platelets and thrombotic microangiopathy (TMA) [1, 3]. Patients of all ages with aHUS have a lifelong, unpredictable risk for clinical manifestations, each potentially leading to end-stage renal disease (ESRD), extrarenal (e.g., neurological, cardiovascular, pulmonary and gastrointestinal) organ damage, and/or death [1, 2, 4]. Recent findings from an observational study in France of 214 patients with aHUS showed that 29 % and 56 % of children and adults, respectively, progressed to ESRD or death within a year of follow-up [2]. Findings from another case series of 273 patients demonstrated that 79 % of aHUS patients have permanent renal damage, require dialysis or die within 3 years of diagnosis [4].

Gene mutations or polymorphisms affecting complement regulators or proteins, including *C3, CFB*, *CFH*, *CFI*, *MCP* (*CD46*) and *CFH-CFHR* genomic rearrangements, or complement factor H autoantibodies are identified in approximately 65–70 % of aHUS patients [2, 4, 5]. However, evidence of complement gene mutations, polymorphisms and/or autoantibodies is not required for the diagnosis of aHUS [6, 7]. Additionally, components of the coagulation and other pathways can modulate complement activation—potentially pathogenic mutations or polymorphisms in genes encoding thrombomodulin (*THBD, CD141)*, diacylglycerol kinase ε (*DGKE)* and plasminogen (*PLG*) [8–10] have been identified in small numbers of patients.

Eculizumab (Soliris®, Alexion Pharmaceuticals, Inc., Cheshire, CT, USA) is a terminal complement inhibitor that is approved for the treatment of aHUS [11, 12]. Eculizumab initially was shown to be effective and well tolerated in two pivotal prospective studies in primarily adult populations of aHUS with evidence of progressing TMA and with long disease duration and chronic kidney disease [13, 14], as well as in a retrospective analysis of pediatric patients with aHUS [15]; these studies led to marketing authorization of eculizumab. Later, a larger prospective study in an exclusively adult population [16] and the first prospective study in a pediatric population [17] also supported the efficacy and safety of eculizumab.

Patient registries have been identified as tools to characterize the natural history of a disease, to evaluate clinical therapies, to monitor drug safety and to measure quality of care [18, 19]. A single, global registry with multiple participating centers worldwide can optimize patient enrollment, which is particularly important for ultra-rare disease states like aHUS. Data generated from an aHUS registry with maximal patient and physician participation can advance epidemiological characterization and inform scientific inquiry and discourse on important issues, such as natural history, systemic manifestations, genotype/phenotype correlations, optimal patient management and long-term treatment outcomes. Also, a global registry provides opportunity for successful partnership between worldwide academia and industry, driven by the shared goal of improving outcomes for patients with aHUS.

Initiated in April 2012, the observational, noninterventional, multicenter, global aHUS Registry has been designed to prospectively collect demographic, disease characteristic and treatment information for all eligible patients with aHUS, regardless of treatment received. The industry-sponsored Registry also fulfills postmarketing regulatory requirements to provide long-term follow-up on patients treated with eculizumab. This current report describes the methodology for the development of the global aHUS Registry, and presents baseline demographic and disease characteristics, as well as data on treatment of aHUS in the clinical practice setting.

## Methods

The global aHUS Registry (US National Institutes of Health www.ClinicalTrials.gov Identifier NCT01522183) was initiated in April 2012 with the support of Alexion Pharmaceuticals, Inc. The objectives of the aHUS Registry are as follows: 1) to assess the long-term effects of aHUS, including clinical outcomes such as TMA complications, and morbidity and mortality in aHUS patients receiving eculizumab treatment or other disease management; and 2) to collect and evaluate safety and effectiveness data specific to the use of eculizumab in aHUS patients.

Scientific oversight, governance and coordination are provided by an independent scientific advisory board (SAB), whose members offer expertise in key specialties related to management of aHUS (e.g., adult and pediatric nephrologists, hematologists, and/or transplant nephrologists/surgeons) and also include representation from Alexion Pharmaceuticals, Inc. The term of SAB membership is 2 years. A member may serve more than one term and also may terminate their membership at any time by notifying the SAB Chairperson.

The SAB coordinates development of scientific publications, including advising on analyses and scientific questions of interest, providing feedback on publication goals and logistics, contributing to the publication plan, establishing criteria for review/approval of external requests for analyses and publications, reviewing publication drafts and counseling individuals who publish data collected from the aHUS Registry. Registry participants and nonparticipating physicians may request data access or specific analyses by submitting a concept sheet or contacting the SAB through the Registry website (when available) for evaluation.

The protocol was approved by the institutional review board at each participating center or by an independent ethics committee, where required, and was conducted in accordance with International Conference on Harmonisation Good Clinical Practice Guidelines and the Declaration of Helsinki. All patients provided written informed consent before study participation.

The forthcoming global aHUS Registry website will be programmed and maintained by a vendor specializing in the development of web-based electronic data collection systems. Investigators will have access to the secure website of the aHUS Registry for entering and accessing patient data online, which will then be stored at a secure and confidential location. Physicians will be able to access the aHUS Registry website at any time for the following purposes: entering and editing data, accessing important documents (frequently asked questions, reminders, etc.), responding to online queries, accessing posted news items, sending/responding to messages, posing questions and accessing standard reports, including enrollment statistics and site-specific patient data reports.

Written informed consent is provided by patients or their parents/guardians, as deemed applicable by institutional review boards and/or independent ethics committees. Clinicians were encouraged to enroll patients of all ages who have already received a clinical diagnosis of aHUS. Diagnosis of aHUS is not performed as part of the Registry protocol. Patients are not required to have an identified complement gene mutation or factor H autoantibody, nor are they required to have previous or ongoing treatment with eculizumab. Individuals with evidence of Shiga toxin-producing *Escherichia coli* infection or with ADAMTS13 activity ≤5 % (the level consistent with a diagnosis of thrombotic thrombocytopenic purpura) are excluded.

All necessary disease history information will be gathered from the patient’s medical records. During enrollment and every 6 months thereafter, the following data are collected as available: patient demographics, medical and disease history, symptomatology, appropriate laboratory results (including those from genetic tests), TMA complications, associated treatments and concomitant medications, clinical and patient-reported outcomes, safety of eculizumab and information regarding treatment or disease management. During each assessment period, physicians will note changes in a patient’s clinical status since the last assessment period. Symptoms and signs listed on the case report forms are renal (edema, hypertension, proteinuria), gastrointestinal (liver necrosis, hepatitis, pancreatitis, diabetes mellitus), cardiovascular (cardiac insufficiency/failure, vasculopathy/atherosclerosis, tachycardia), central nervous system (confusion, focal neurological deficit, ocular defects, headache) and pulmonary (hemorrhage, edema, shortness of breath). Clinicians have the option to list additional symptoms for these organ systems. Occurrences of multisystemic symptoms (i.e., symptoms/signs categorized under more than one organ system) also are recorded. Baseline values were defined as those collected at time of Registry enrollment or before the first dose of eculizumab (for patients who received eculizumab). Safety evaluations included adverse events and/or side effects associated with eculizumab treatment and other management strategies and measurement of human anti-human antibodies. Adverse events of interest included meningococcal and other serious infections, malignancy, renal impairment, hepatic impairment, sepsis and infusion reactions.

The data cutoff for this analysis was September 30, 2014. Patients with all of the following data were included in the current analysis of enrollment characteristics: 1) date of birth, gender, Registry enrollment date; 2) knowledge of treatment with eculizumab or no previous eculizumab treatment; and 3) for eculizumab-treated patients, date of first eculizumab dose. Patients were stratified by age at enrollment into the Registry.

Descriptive statistical analysis was performed to summarize demographics and clinical data. Proportions were calculated for categorical measures and summary statistics (n, mean, standard deviation and median) for continuous measures. All data analyses were performed in a validated statistical programming environment using SAS® statistical software version 9.2.

## Results

As of September 30, 2014, 195 clinical sites in 17 countries were open to enrollment and a total of 516 patients were enrolled. Table 1 summarizes baseline demographic characteristics among pediatric (*n* = 201; 39.0 %) and adult (*n* = 315; 61.0 %) patients, most of whom were Caucasian and from the United States or Western Europe.

**Table 1:** Patient Demographics in the Global aHUS Registry

Characteristic	Pediatric patients^a^ (*n* = 201)	Adult patients^a^ (*n* = 315)	Total (*N* = 516)
Age at enrollment, years			
Mean (SD)	8.2 (4.9)	39.7 (15.4)	27.4 (19.7)
Median (range)	8.3 (0.0–17.9)	38.3 (18.0–81.8)	24.3 (0.0–81.8)
Age category at time of enrollment, *n* (%)			
<2	22 (10.9)	—	22 (4.3)
2 to <5	41 (20.4)	—	41 (7.9)
5 to <12	82 (40.8)	—	82 (15.9)
12 to <18	56 (27.9)	—	56 (10.9)
18 to <30	—	102 (32.4)	102 (19.8)
30 to <40	—	73 (23.2)	73 (14.1)
40 to <50	—	61 (19.4)	61 (11.8)
≥50	—	79 (25.1)	79 (15.3)
Female, *n* (%)	81 (40.3)	194 (61.6)	275 (53.3)
Race, *n* (%)			
Caucasian	162 (80.6)	285 (90.5)	447 (86.6)
Other	27 (13.4)	10 (3.2)	37 (7.2)
Black	5 (2.5)	17 (5.4)	22 (4.3)
Asian	7 (3.5)	3 (1.0)	10 (1.9)
Country at time of enrollment, *n* (%)			
United States	31 (15.4)	77 (24.4)	108 (20.9)
Germany	32 (15.9)	50 (15.9)	82 (15.9)
United Kingdom	33 (16.4)	33 (10.5)	66 (12.8)
Italy	20 (10.0)	45 (14.3)	65 (12.6)
Spain	24 (11.9)	13 (4.1)	37 (7.2)
France	3 (1.5)	25 (7.9)	28 (5.4)
Russia	17 (8.5)	10 (3.2)	27 (5.2)
Belgium	5 (2.5)	22 (7.0)	27 (5.2)
Australia	2 (1.0)	24 (7.6)	26 (5.0)
Israel	18 (9.0)	2 (0.6)	20 (3.9)
Austria	1 (0.5)	9 (2.9)	10 (1.9)
Canada	6 (3.0)	1 (0.3)	7 (1.4)
United Arab Emirates	6 (3.0)	0 (0.0)	6 (1.2)
Sweden	3 (1.5)	1 (0.3)	4 (0.8)
Switzerland	0 (0.0)	2 (0.6)	2 (0.4)
Finland	0 (0.0)	1 (0.3)	1 (0.2)
Year of enrollment			
2012	1 (0.5)	20 (6.3)	21 (4.1)
2013	140 (69.7)	189 (60.0)	329 (63.8)
2014^b^	60 (29.9)	106 (33.7)	166 (32.2)
Deceased, *n* (%)^c^	1 (0.5)	14 (4.4)	15 (2.9)

*aHUS* atypical hemolytic uremic syndrome, *SD* standard deviation

^a^Categorized by age at enrollment in the Registry

^b^Through September 30, 2014

^c^Each of these causes of death were reported in 1 patient: acute myeloid leukemia; *Aspergillus* infection; cardiomyopathy; continuous gastrointestinal bleeding; gastrointestinal perforation; liver metastases; malignant cardiac arrhythmias; protracted cardiogenic shock and ventricular tachycardia; renal failure in addition to aHUS and pancreatic cancer; respiratory arrest probably due to cytomegalovirus infection; respiratory failure due to pneumonia; respiratory failure secondary to bleomycin lung injury; serious infection leading to multiple organ failure; unknown; withdrawal from dialysis and prostate cancer

Clinical characteristics of patients in the global aHUS Registry are shown in Table 2. Pediatric and adult patients were a mean of 4.3 and 34.5 years of age, respectively, at aHUS diagnosis. Forty-six adult patients (14.6 %) had aHUS symptom onset at <18 years of age, and 41 (13.0 %) were diagnosed with aHUS at <18 years of age. Overall, 99 patients (19.2 %) reported a family history of aHUS. Although 307 patients (59.5 %) reported a history of dialysis, only 101 (19.6 %) underwent 1 or more renal transplantations, which occurred more frequently in adult compared with pediatric patients. Most patients had a history of plasma exchange/plasma infusion (PE/PI) and/or dialysis use, although these management strategies were used more commonly for adult patients compared with pediatric patients. Prior to Registry enrollment, 274 patients (53.1 %) were treated with eculizumab. Overall, 305 total patients (59.1 %) enrolled in the Registry, including 117 pediatric patients (58.2 %) and 188 adult patients (59.7 %), have been treated with eculizumab.

**Table 2:** Clinical Characteristics of Patients in the Global aHUS Registry

Characteristic	Pediatric patients (*n* = 201)^a^	Adult patients (*n* = 315)^a^	Total (*N* = 516)^a^
Age at initial symptoms, years			
*n*	198	310	508
Mean (SD)	4.1 (3.9)	33.6 (18.3)	22.1 (20.4)
Median (range)	2.9 (0.0–16.3)	31.1 (0.1–81.8)	19.2 (0.0–81.8)
Age categories, *n* (%)			
<2	87 (43.9)	12 (3.9)	99 (19.5)
2 to <5	40 (20.2)	9 (2.9)	49 (9.6)
5 to <12	60 (30.3)	14 (4.5)	74 (14.6)
12 to <18	11 (5.6)	11 (3.5)	22 (4.3)
18 to <30	—	95 (30.6)	95 (18.7)
30 to <40	—	71 (22.9)	71 (14.0)
40 to <50	—	34 (11.0)	34 (6.7)
≥50	—	64 (20.6)	64 (12.6)
Age at diagnosis, years			
*n*	201	313	514
Mean (SD)	4.3 (4.0)	34.5 (17.9)	22.7 (20.5)
Median (range)	3.1 (0.0–16.3)	31.5 (0.1–81.8)	19.7 (0.0–81.8)
Age categories, *n* (%)			
<2	86 (42.8)	10 (3.2)	96 (18.7)
2 to <5	39 (19.4)	6 (1.9)	45 (8.8)
5 to <12	63 (31.3)	11 (3.5)	74 (14.4)
12 to <18	13 (6.5)	14 (4.5)	27 (5.3)
18 to <30	—	98 (31.3)	98 (19.1)
30 to <40	—	71 (22.7)	71 (13.8)
40 to <50	—	35 (11.2)	35 (6.8)
≥50	—	68 (21.7)	68 (13.2)
Disease duration from diagnosis to enrollment, years			
*n*	201	313	514
Mean (SD)	4.0 (4.1)	5.2 (7.8)	4.7 (6.6)
Median (range)	2.4 (0.0–17.1)	1.6 (0.0–50.6)	1.9 (0.0–50.6)
Family history of aHUS, *n* (%)	41 (20.4)	58 (18.4)	99 (19.2)
History of PE/PI, *n* (%)	107 (53.2)	204 (64.8)	311 (60.3)
History of dialysis, *n* (%)	107 (53.2)	200 (63.5)	307 (59.5)
History of kidney transplantation, *n* (%)	25 (12.4)	76 (24.1)	101 (19.6)
History of eculizumab use prior to enrollment, *n* (%)	106 (52.7)	168 (53.3)	274 (53.1)

*aHUS* atypical hemolytic uremic syndrome, *PE/PI* plasma exchange/plasma infusion, *SD* standard deviation

^a^For each parameter, percentages were calculated based on the number of patients with non-missing observations in each group

## Discussion

This report describes the initial enrollment, pattern of global participation and types of data gathered in the industry-sponsored global aHUS Registry, and outlines baseline demographic and clinical characteristics of current participants. While aHUS and TMA registries have been established previously [4, 20, 21], the global aHUS Registry is the largest and the only one to include aHUS patients both treated and not treated with eculizumab. Overall, patients were diagnosed with aHUS soon after presenting with initial symptoms. While historically, aHUS was thought to manifest primarily in children [1], 53 % of current Registry participants were adults at diagnosis. This is similar to findings from a recent aHUS case series, which demonstrated that 58 % of cases in France occur in patients older than 18 years [2]. However, the age distribution of participants in the Registry will be influenced by the focus of sites (i.e., adult or pediatric) that are currently open.

Historically, PE/PI has been used to manage aHUS [22] by supplying functional natural regulators of complement, thus temporarily maintaining hematologic parameters [1, 22, 23]. Eculizumab, the only approved treatment for aHUS [11, 12], has been shown to inhibit complement-mediated TMA and is well tolerated [13, 14]. Several current guidelines recommend the immediate initiation of eculizumab for pediatric and adult patients once a diagnosis of aHUS is made [6, 7, 24, 25]. Thus far, 59.1 % of patients in the Registry have been treated with eculizumab. As the Registry grows, it will become more feasible to evaluate the effects of eculizumab therapy compared with patients who are managed with PE/PI or other options, in a larger population than was included in the prospective clinical trial program for eculizumab [13, 14, 16, 17]. In the future, it will be of particular interest to evaluate longer-term outcomes to help elucidate optimal treatment with eculizumab.

Limitations of the Registry include possible underreporting of outcomes, missing data, and/or inadequate follow-up. Robustness of data gathered depends on the quality of data entry, number of aHUS patients who enroll, the diversity of their demographic and disease characteristics, including age and disease duration, and retention of recruited patients. Thus far, enrolled patients have had a mean of 5-year histories of aHUS before Registry enrollment. Future enrollment of patients who are newly diagnosed with aHUS will allow for analysis of more contemporary treatment and management approaches. Other limitations include the potential for varying interpretation of baseline disease characteristics by enrolling physicians. Increased clinician participation and collection of complete information will optimize the quality of Registry results and inform forthcoming analyses.

Future analyses from the global aHUS Registry will be conducted to increase understanding of the natural history and progression of the disease. Data gathered during the minimum 5-year follow-up period are collected from clinicians worldwide and will be valuable to help describe aHUS presentation, progression with or without eculizumab therapy, and patient outcomes. Thus far, observational studies in aHUS patients have led to varying conclusions regarding the effect of genetic background on prognosis [2, 4]. Although complement gene mutations, polymorphisms or factor H autoantibodies typically are identified in up to 70 % of patients [2, 4, 26], they are not required for a diagnosis of aHUS [6, 7]. As part of ongoing research efforts, future analyses of the global aHUS Registry may also help to determine the relative degree to which genetic and other patient factors (e.g., age, gender and ethnicity) predict the development and clinical course of complement-mediated TMA complications. Atypical hemolytic uremic syndrome is typically characterized by renal involvement [1]; however, the medical literature increasingly is reporting extrarenal complications of the disease [4, 27], including cardiovascular, neurological, pulmonary, and gastrointestinal. Future analyses will help to better characterize the development and progression of extrarenal and multisystemic complications. Several conditions that may potentially further increase complement activity are known to uncover an underlying diagnosis of aHUS and place patients at increased risk for renal and/or extrarenal organ damage [1, 28, 29]. The Registry is open to all patients with a clinical history of aHUS, including those with these types of comorbid conditions that have been associated with TMA and/or aHUS, including autoimmune disease, malignancy, malignant hypertension, pregnancy-associated conditions of preeclampsia and HELLP (hemolysis, elevated liver enzymes, low platelet count) syndrome, and scleroderma [4, 7, 30, 31]. Inclusion of these patients may allow for further characterization of the relationship between these conditions and aHUS in future analyses. Finally, because enrollment in the Registry is not contingent upon receipt of eculizumab, data comparing the long-term outcomes of patients treated with eculizumab and patients managed with supportive therapies, including PE/PI, may be included in future analyses.

## Conclusions

Finally, data derived from the aHUS Registry may also be valuable in defining strategies for optimization of patient care. With inclusion of both treated and untreated patients, the Registry will make it possible to characterize the long-term effectiveness and safety profile of eculizumab across a diverse patient population.

## Abbreviations

aHUS: atypical hemolytic uremic syndrome
ESRD: end-stage renal disease
HELLP: hemolysis, elevated liver enzymes, low platelet count
PE/PI: plasma exchange/plasma infusion
SAB: scientific advisory board
SD: standard deviation
TMA: thrombotic microangiopathy
